# Throwing light on dark diversity of vascular plants in China: predicting the distribution of dark and threatened species under global climate change

**DOI:** 10.7717/peerj.6731

**Published:** 2019-04-09

**Authors:** Lili Tang, Runxi Wang, Kate S. He, Cong Shi, Tong Yang, Yaping Huang, Pufan Zheng, Fuchen Shi

**Affiliations:** 1 College of Life Sciences, NanKai University, Tianjin, China; 2 Department of Biological Sciences, Murray State University, Murray, KY, USA; 3 School of Environment and Energy, Shenzhen Graduate School, Peking University, Shenzhen, China

**Keywords:** Dark diversity, Global climate change, Maximum entropy, Species distribution, Threatened plants

## Abstract

**Background:**

As global climate change accelerates, ecologists and conservationists are increasingly investigating changes in biodiversity and predicting species distribution based on species observed at sites, but rarely consider those plant species that could potentially inhabit but are absent from these areas (i.e., the dark diversity and its distribution). Here, we estimated the dark diversity of vascular plants in China and picked up threatened dark species from the result, and applied maximum entropy (MaxEnt) model to project current and future distributions of those dark species in their potential regions (those regions that have these dark species).

**Methods:**

We used the Beals probability index to estimate dark diversity in China based on available species distribution information and explored which environmental variables had significant impacts on dark diversity by incorporating bioclimatic data into the random forest (RF) model. We collected occurrence data of threatened dark species (*Eucommia ulmoides*, *Liriodendron chinense*, *Phoebe bournei*, *Fagus longipetiolata*, *Amentotaxus argotaenia*, and *Cathaya argyrophylla*) and related bioclimatic information that can be used to predict their distributions. In addition, we used MaxEnt modeling to project their distributions in suitable areas under future (2050 and 2070) climate change scenarios.

**Results:**

We found that every study region’s dark diversity was lower than its observed species richness. In these areas, their numbers of dark species are ranging from 0 to 215, with a generally increasing trend from western regions to the east. RF results showed that temperature variables had a more significant effect on dark diversity than those associated with precipitation. The results of MaxEnt modeling showed that most threatened dark species were climatically suitable in their potential regions from current to 2070.

**Discussions:**

The results of this study provide the first ever dark diversity patterns concentrated in China, even though it was estimated at the provincial scale. A combination of dark diversity and MaxEnt modeling is an effective way to shed light on the species that make up the dark diversity, such as projecting the distribution of specific dark species under global climate change. Besides, the combination of dark diversity and species distribution models (SDMs) may also be of value for ex situ conservation, ecological restoration, and species invasion prevention in the future.

## Introduction

Biodiversity is a fundamental and central topic in ecology and conservation. In recent years, ecologists have been increasingly investigating the effects of climate change on biodiversity and species’ distribution (Dawson et al., 2011; Guillera-Arroita et al., 2015; Gomes et al., 2018). Most of these studies relied on the observed species records from field sites to predict the response of species to climate change and to monitor biodiversity changes (Cárdenas et al., 2011; Dawson et al., 2011; Turner, 2014; Storkey et al., 2015; Gray et al., 2016). However, the observed species do not reflect the complete habitat-specific species pool of a site as some species will remain undetected due to limited sampling efforts and resources of the investigators and the timing of sampling (Pärtel, Szava-Kovats & Zobel, 2011). For example, rare species and species with very short lifespan can be overlooked easily. That is especially true when sampling units are large in space and sparse in time. This particular set of species that belong to a particular species pool but are not locally present, or that are absent from a community but have the potential to establish are known as “dark diversity” (Lewis, Szava-Kovats & Pärtel, 2016; Pärtel, Szava-Kovats & Zobel, 2011, 2013).

Since dark diversity was proposed (Pärtel, Szava-Kovats & Zobel, 2011), there have been rapid developments regarding its estimating methods. Ellenberg indicator values (Ellenberg et al., 1991; Hill et al., 1999; Pärtel et al., 1996) and Beals probability index (Beals, 1984) are the most common methods to estimate dark diversity. In addition, SDMs are also used to complement dark diversity estimations (De Bello et al., 2016; Lewis, Szava-Kovats & Pärtel, 2016; Ronk et al., 2016). Ellenberg indicator values directly estimate dark diversity or species pool size, and it can only be used when there is sufficient information on the habitat requirements of species (De Bello et al., 2016; Lewis, Szava-Kovats & Pärtel, 2016; Ronk et al., 2016). By contrast, the Beals probability index uses the geographical distribution or co-occurrence patterns of species as a proxy of their ecological requirements (De Bello et al., 2016; Lewis, Szava-Kovats & Pärtel, 2016; Moeslund et al., 2016; Riibak et al., 2015; Ronk et al., 2016, 2017; Ronk, Szava-Kovats & Pärtel, 2015). Moreover, comparative studies have indicated that this proxy gives accurate estimates of dark diversity for macro-scale ecological communities (Lewis, Szava-Kovats & Pärtel, 2016; Ronk et al., 2016).

To date, most research on dark diversity has been conducted in Europe (Kasari et al., 2016; Lewis et al., 2017; Ronk, Szava-Kovats & Pärtel, 2015), and those studies have shown that dispersal limitation and stress-tolerance play essential roles in shaping the patterns of observed and dark diversity (Riibak et al., 2015). However, the impacts of future climate change are largely unknown on shaping the patterns of observed and dark diversity in those studies. Furthermore, most studies have focused on mapping dark diversity at macro-scale and comparing methods to estimate dark diversity but ignored the specific information on the identity of species that constitute dark diversity. For example, questions such as whether the dark diversity dataset includes any threatened species and what would happen to their potential distribution with global climate change have not yet been answered.

In this study, we tried to seperate threatened species from dark diversity pool and to explore how their distributions shift in the future in China using the species distribution model. We proposed three fundamental questions: (1) What is the level and distribution of dark diversity in China, and does the dark diversity include threatened species? (2) Which environmental variables affect dark species distributions the most? (3) Will the potential areas of dark (particularly threatened) plants remain suitable in the future under global climate change? To address these questions, we calculated the Beals probability index using previously complied vascular plants datasets at the macro-scale to investigate the level of dark diversity in China. We also used random forest (RF) model to assess the contribution of a number of variables to dark diversity. Lastly, we performed maximum entropy (MaxEnt) modeling to project changes in the distribution of threatened dark species under climate scenarios in the years of 2050 and 2070. The results of this study could provide novel information on dark diversity in China, as this has never been investigated before, to our best knowledge. Further, information on dark diversity at a regional scale could have significant implications on conservation efforts, including ecological restoration and invasion risk assessment.

## Materials and Methods

### Species distribution data and environmental variables

**Figure 1:**
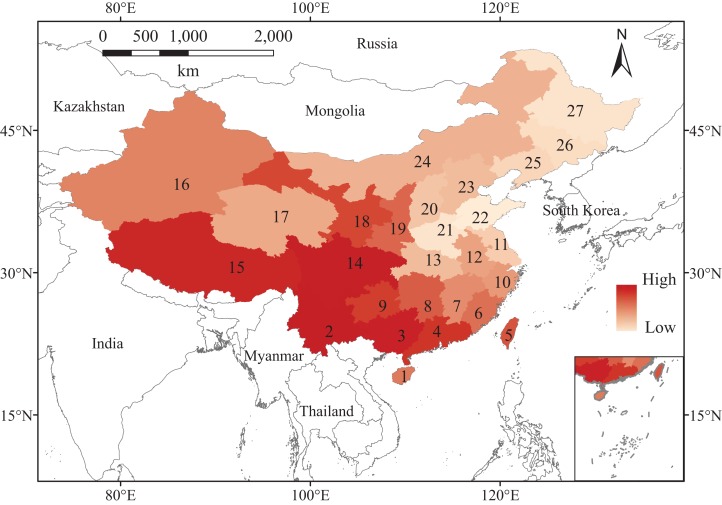
Map of China, showing the observed species richness for vascular plants (dark and light represent high and low richness levels, respectively). The provinces are numbered and labeled as follow: Hainan (1), Yunnan (2), Guangxi (3), Guangdong including Hongkong and Macau (4), Taiwan (5), Fujian (6), Jiangxi (7), Hunan (8), Guizhou (9), Zhejiang (10), Jiangsu including Shanghai (11), Anhui (12), Hubei (13), Sichuan including Chongqing (14), Xizang (15), Xinjiang (16), Qinghai (17), Gansu including Ningxia (18), Shaanxi (19), Shanxi (20), Henan (21), Shandong (22), Hebei including Beijing and Tianjin (23), Neimenggu (24), Liaoning (25), Jilin (26), Heilongjiang (27).

This study covered the entire area of China. We retrieved vascular plants occurrence data in each province and municipality of China from the Flora of China (http://frps.eflora.cn/) and merged data of municipalities into their nearest provinces, so we got 27 records in total (Fig. 1). In comparison with the data applied to the study by Pärtel, Szava-Kovats & Zobel (2011), derived from a global map and at the scale of 100 x 100 km, although our data are at larger scale, the observed species lists are much more complete, especially for that of Northern China, which was considered difficult to access (Kier et al., 2005). We constructed an individual species dataset for them to show which plants were present or absent. Although the floristic data have been collected since 1959, we assume that they are still relevant because plants, particularly those in the temperate forest, respond relatively slowly to environmental changes (Franklin et al., 2016; Menéndez et al., 2006). We only included plant data at the species level, and we merged all subspecies. We also excluded all cultivated species that were not considered a natural part of the vegetation based on their life history and dispersal traits. Synonyms and conservation status such as threatened species were checked using The Plant List (http://www.theplantlist.org/) and The IUCN Red List of Threatened Species (http://www.iucnredlist.org/). This observed species richness (number of species) distribution shows a latitudinal trend, increasing from north to south in China (Fig. 1).

Climatic variables, such as precipitation and temperature have a significant impact on species’ distribution, particularly at large spatial scales and over long timeframes (Beer et al., 2010; Kelly & Goulden, 2008; Loarie et al., 2009; Williams, Jackson & Kutzbach, 2007). We extracted 19 bioclimatic variables (BIO1–BIO19; Table S1) in years of current, 2050 and 2070, respectively, from Worldclim (resolution, 2.5 s; www.Worldclim.org/). As for the variables in 2050 and 2070, we selected those under representative concentration pathway 4.5 (RCP4.5) and based on the CCSM4 global climate model (GLM). There are four RCPs, ranging from very high (RCP8.5, the temperature will increase to 3.7 ± 0.7 °C) through to deficient (RCP2.6, the temperature will rise to 1.0 ± 0.4 °C) future concentrations (Collins et al., 2013). Based on current status of global warming and the goal that hold average temperature increase to well below 2 °C (IPCC, 2018), in this study, we chose RCP4.5 scenario as the future climate scenario. In addition, according to the projection by Ying (2012), until the year of 2018, the CCSM4 has been showing as the more accurate model than the other 16 GLMs under RCP4.5 scenarios, so in this study, the future climate projections are based on the CCSM4 climate model. Current and future bioclimatic projection data were used in the MaxEnt model to predict habitat suitability of six threatened dark species that will be detailed in the following sections.

### Estimation of dark diversity and community completeness

To obtain the best possible estimates of dark diversity in China, we used Beals probability index (Beals, 1984), as recommended by Lewis, Szava-Kovats & Pärtel (2016). This index can be used to estimate the probability of a species occurring in a particular region based on its co-occurrence within other regions and was calculated using the package “vegan” (Oksanen et al., 2016) in R ver. 3.3.1 (R Development Core Team, 2016).

The Beals index is defined as:

$${P_{ij}} = {1 \over {{S_i} - {I_{ij}}}}\sum\limits_{k \ne j} {{{{N_{jk}}{I_{ik}}} \over {{N_k}}}}$$

where *P*_*ij*_ is the probability that species *j* will occur at community *i*, *S*_*i*_ is the number of species at community *i*, *I*_*ij*_ is the incidence (0, 1) of species *j* at community *i*. *N*_*jk*_ is the number of joint occurrence of species *j* and *k* (*k* ≠ *j*) at community *i*, *I*_*ik*_ is the incidence (0, 1) of species *k* at community *i*, and *N*_*k*_ is the number of occurrences of species *k* (for further details, see Münzbergová & Herben (2004) and Lewis, Szava-Kovats & Pärtel (2016)).

The probability of occurrence varies among species, regions and depends on the frequencies of species in a particular assemblage. Each species was assigned an individual probability threshold, which was a quantile calculated from a user-defined probability. Based on the comparison of the dark diversity from different probabilities (1%, 5%, 10%) (Table S2), to explore the broader possible species pool in China, it was defined 1% in all regions here. For each area, a species was included in the dark diversity when it was absent from a target region and its occurrence probability was higher than its threshold value.

In addition, we calculated the community completeness index for each region using ln (observed richness/dark diversity) (Pärtel, Szava-Kovats & Zobel, 2013).

### Assessing variables that affect dark diversity

We used RF to assess the contribution of bioclimatic variables toward the dark diversity. The RF technique estimates the importance of a predictive variable by evaluating the Out-of-bag (OOB) error increase (Mutanga, Adam & Cho, 2012). In other words, the decrease of prediction accuracy, represented as the percentage of increased mean square error (% IncMSE) when OOB data for the specific variable is switched while all the other variables remained (Mutanga, Adam & Cho, 2012). Here, we extracted a matrix of 3,844 spot records covering the whole area of China, with their 19 current bioclimatic data, and applied it to RF, where their dark diversity values at the regional level are treated as the response variable.

Random forest is a machine learning approach based on classification and regression trees (CART; Breiman, 2001). The RF model-building process is similar to that of CART; only it combines numerous independent trees to reach a final decision (for further details, see Wiesmeier et al. (2011) and Da Silva Chagas et al. (2016)). We estimated the relative importance of each variable in the RF model using OOB randomly selected data. The mean square error (MSE) was calculated as:

$${\rm MS}{{\rm E}_{{\rm OBB}}} = \displaystyle{1 \over N}\mathop \sum \limits_{i = 1}^n {\left( {{Z_i} - \hat Z_i^{{\rm OBB}}} \right)^2}$$

where *Z*_*i*_ is the measured value of the variable and $\hat Z_i^{{\rm OBB}}$ is the average of all OBB predictions. The MSE_OBB_ is normalized as it depends on the unit of the response variable.

Random forest modeling was implemented by R ver. 3.3.1 (R Development Core Team, 2016), using the “RF” package (Liaw & Wiener, 2002). The number of trees (ntree) was 500 and the number of randomly selected predictor variables at each node (mtry) was three.

### Predicting the distribution of rare dark species with future climate change

We used MaxEnt modeling (Version 3.4.1) to predict changes in the potential areas of six rare dark plant species under global climate change. MaxEnt is a widely used species distribution algorithm, and many studies have compared it with other SDMs to confirm its predictive ability (Elith & Graham, 2009; Elith, Kearney & Phillips, 2010; Tognelli et al., 2009; Williams et al., 2009). It is used to predict the geographic distributions of species based on incomplete information of species (presence-only datasets) occurrence data and environmental variables by calculating the MaxEnt of species distribution (Phillips, Anderson & Schapire, 2006; Phillips & Dudik, 2008).

In this study, we chose the near threatened, vulnerable, and endemic species*: Eucommia ulmoides*, *Liriodendron chinense, Phoebe bournei, Fagus longipetiolata, Amentotaxus argotaenia*, and *Cathaya argyrophylla* from the threatened dark species based on available data and species’ distribution range: all species have a limited distribution and a small dataset of occurrence sites. In total, we found 187 occurrence sites for these species in relevant references, local flora, and the flora of China, which we included in the MaxEnt model (*A. argotaenia:* 30 sites*, C. argyrophylla*: 22 sites*, E. ulmoides*: 32 sites*, L. chinense*: 35 sites*, F. longipetiolata*: 29 sites*, P. bournei*: 39 sites) (Fig. S1; Table 1). According to the records of the references, geographic coordinate data are within an accuracy of about three km. The 19 bioclimatic variables were filtered out some less important ones by using RF model and removed a few high correlated variables by using correlation coefficient. The correlation coefficient was implemented in R ver. 3.3.1 (R Development Core Team, 2016), using the “raster” package (Hijmans, 2018), the cut-off threshold is 0.80.

**Table 1:** Threatened species among the dark diversity in China.

	Species	Threatened level	Potential area	Life form	Life span	Habitat types	^i^Data source for MaxEnt	Year of data source
1	*Amentotaxus argotaenia*	NT	Anhuai	Tree	Perennial	Forest	1–6	2000, 2001, 2004, 2007, 2014, 2017
2	*Eucommia ulmoides*	NT	Jiangsu (Shanghai)	Tree	Perennial	Forest	7–10	2013, 2014, 2016
3	*Liriodendron chinense*	NT	Jiangsu (Shanghai) & Guangdong (Hongkong, Macao)	Tree	Perennial	Forest	3, 11–18	2003, 2007, 2011, 2014, 2016, 2017
4	*Phoebe bournei*	NT	Anhuai & Jiangsu (Shanghai)	Tree	Perennial	Forest	19	2012
5	*Cathaya argyrophylla*	VU	Hubei	Tree	Perennial	Forest	20–22	1994, 2006, 2016
6	*Fagus longipetiolata*	VU	Jiangsu (Shanghai)	Tree	Perennial	Forest	23, 24	1997, 2008
7	*Cycas taiwaniana*	EN	Guizhou & Hainan	Tree	Perennial	Forest		
8	*Dendrobium officinale*	CR	Guizhou	Tree	Perennial	Forest		

**Note:**

IUCN, International Union for the Conservation of Nature threat levels; NT, near threatened; VU, vulnerable; EN, endangered; CR, critically endangered. Potential area was estimated by dark diversity model.

i Details can be found in the Supplemental Information.

In our model, we split the dataset into two parts: 75% of the occurrence data were used as training set, while the remaining 25% was used as a test set to evaluate the strength of the model. In this model, we set the 10,000 points as the max number of background sampling entire China. As for the small size of our data, we tuned the regularization multipliers (0.1, 0.25, 0.35, 0.5, 0.75, 1, 1.25, 1.5, 1.75, 2, 2.25, 2.5, 2.75, and 3) in MaxEnt model, to find out the best one with the smallest corrected Akaike information criterion (AICc) value (Morales, Fernández & Baca-González, 2017). The AICc was calculated by ENMTOOLS (version 1.4.4) (Warren & Seifert, 2011). The area under the receiver operating characteristic curve (AUC) and omission error (minimum training presence as the logistic threshold) was used to quantify the strength of the current scenario models. AUC values range from zero to one, with the values of ≤0.5 indicating that the model performs worse than a random model and values close to 1 indicating that the model performs better than a random model (Swets, 1988). In this study, we considered values of 0.5–0.6 to represent no discrimination, 0.6–0.7 as unaccepted, 0.7–0.8 as accepted, 0.8–0.9 as excellent, 0.9–1 as outstanding and 1 as perfect (Mischler et al., 2012; Phillips, Anderson & Schapire, 2006). Finally, we used the Jackknife test to evaluate which variable made the greatest contribution to the distribution of each of these six species under the current climatic scenario.

## Results

### Dark diversity and community completeness in China

**Figure 2:**
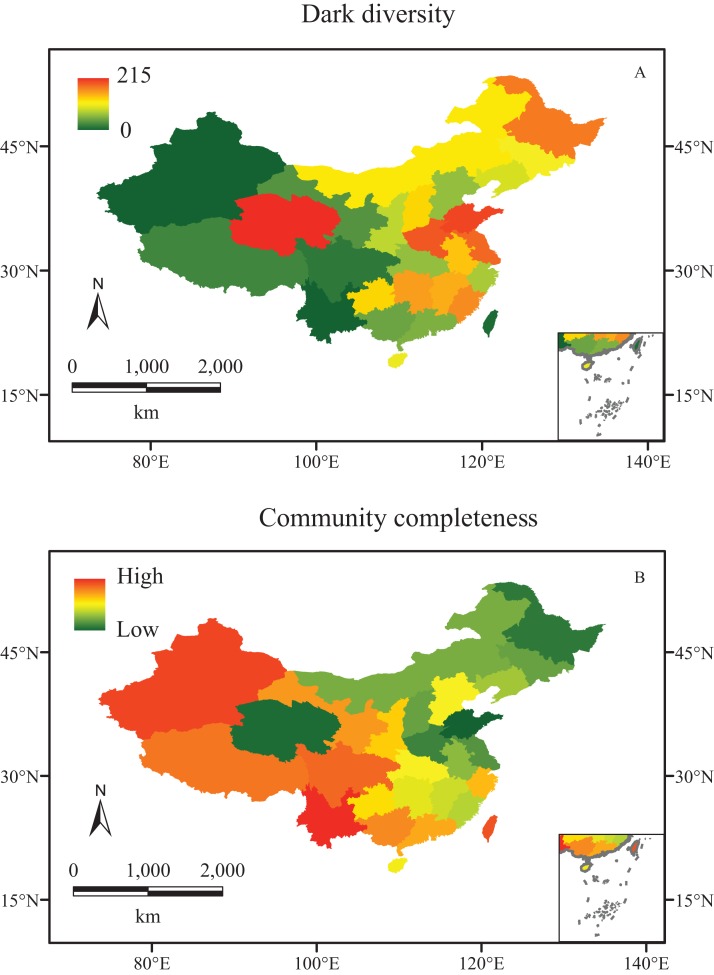
The biodiversity of vascular plant species in China. (A) Dark diversity, and (B) community completeness (ln (observed richness/dark diversity)). Red and green indicate high and low values, respectively.

Different regions in China show various dark diversities, ranging from 0 to 215. In comparison with their observed local richness (from 1,170 to 11,760), their dark diversities are smaller. Meanwhile, the observed richness showed a latitudinal gradient change, increasing from north to south (Fig. 1), while dark diversity exhibited no latitudinal trend, and the values declined from east to west, except for Qinghai. The highest levels of dark diversity were concentrated in three regions: Qinghai (215), the southeast of the northern China plain (Shandong, 159; Henan, 155; Jiangsu including Shanghai, 143), and Heilongjiang (142) (Fig. 2A).

The community completeness indexes showed a similar distribution as their observed richness, almost fully complete in the west communities (except for Qinghai Province), and less complete in the east. In addition, the completeness indexes were lower in the northeast than those in the southeast of China (Fig. 2B).

We identified eight threatened species among the dark diversity, representing a range of threat levels (Table 1).

### Explanatory variables for dark species

**Figure 3:**
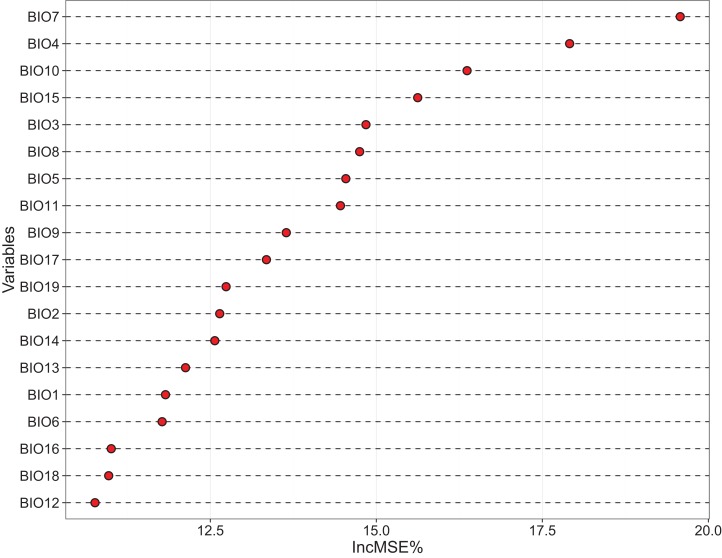
Variables importance derived from random forest models showed by increase in MSE (%). BIO 1–BIO 11 are the variables associated with temperature, while BIO 12–BIO 19 are the variables associated with precipitation. In this figure, most of temperature variables are gain the high % IncMSE.

The relative importance of the 19 bioclimatic variables regarding dark diversity in China, as assessed using RF, is shown in Fig. 3. We found that variables associated with temperature showed more importance than those associated with precipitation (Fig. 3). However, together these variables only explained 71.72% of the variance, thus few unknown factors remain. The most important variables were Temperature annual range (BIO7) and Temperature seasonality (BIO4), Mean temperature of warmest quarter (BIO10), and Precipitation seasonality (BIO15). Other significant variables were Isothermality (BIO3), Mean temperature of wettest quarter (BIO8), Max temperature of warmest month (BIO5), Mean temperature of coldest quarter (BIO11), Mean temperature of driest quarter (BIO9), Precipitation of driest quarter (BIO17), Precipitation of coldest quarter (BIO19), Mean diurnal range (BIO2), and Precipitation of driest month (BIO14). These variables were filtered to use in MaxEnt projection firstly.

Then according to the results of cross-correlation (Fig. S2), we selected seven bioclimatic variables used in the MaxEnt model for current climate scenario (Fig. S2), and the contribution of them differed between six species (Table 2). For these six species, BIO14 in current bioclimatic scenarios was the most important variable when projected their distribution, BIO5 also showed its significance to project the species distribution (Table 2). For the 2050 and 2070 climate scenarios, we selected six bioclimatic variables used in MaxEnt (Fig. S2).

**Table 2:** Summary of the contribution of the bioclimatic variables used in the MaxEnt model and the omission rate, AICc, and AUC values for the model.

Species	Contribution to MaxEnt models (%)							Betamultiplier	AICc	Fractional predicted area	Omission rate	AUC
	BIO2	BIO3	BIO5	BIO7	BIO11	BIO14	BIO15					
*Amentotaxus argotaenia*	0.02	0	21.09	9.23	6.74	62.16	0.77	0.5	1,741.1	0.175	0	0.971
*Cathaya argyrophylla*	1.86	2.25	30.12	1.63	4.8	59.33	0	2.25	603.46	0.036	0	0.995
*Eucommia ulmoides*	0.75	6.53	7.26	13.95	38.8	30.65	2.05	2	966.64	0.172	0	0.94
*Fagus longipetiolata*	0.41	1.24	30.2	1.01	1.45	65.14	0.55	1	826.84	0.076	0.286	0.956
*Liriodendron chinense*	6.84	0	13.56	1.97	12.92	64.45	0.26	1.5	1,033.54	0.198	0	0.948
*Phoebe bournei*	0.72	0.01	8.28	8.09	0.28	81.16	1.46	0.75	1,122.42	0.07	0.222	0.953

**Note:**

According to omission rate and AUC values, all the models were considered outstanding. For six trees, BIO14 was the most important variables when projected their distributions under current climatic scenarios.

### Distribution of dark threatened species in the future

All the models under current bioclimatic scenarios were considered outstanding according to their omission error, AUC and AICc values (Table 2), suggesting that they could accurately predict species distributions.

**Figure 4:**
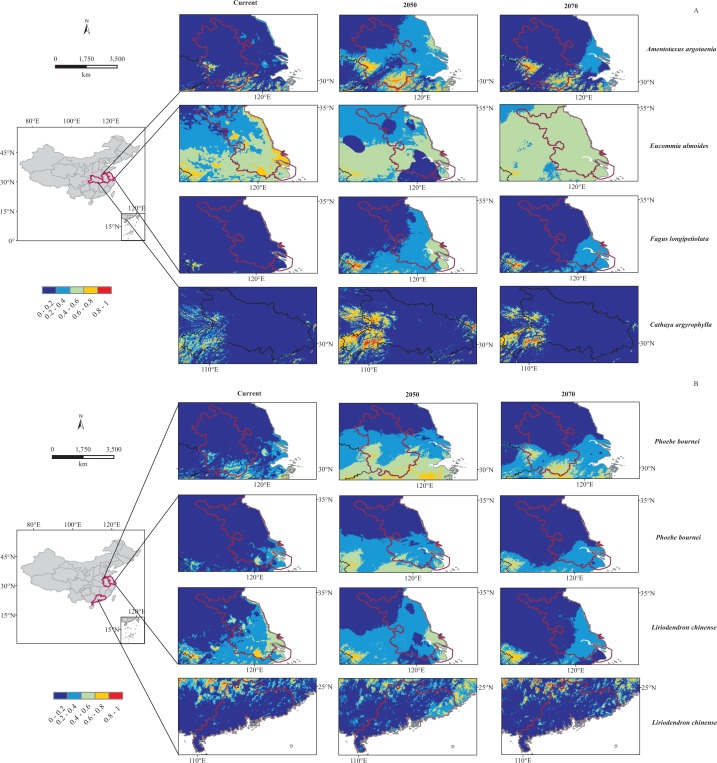
Predicted distribution of six dark species under current and future (2050, 2070) bioclimatic scenarios in potential regions. (A) For *Amentotaxus argotaenia*, *Eucommia ulmoides*, *Cathaya argyrophylla*, *Fagus longipetiolata*. (B) For *Liriodendron chinense* and *Phoebe bournei*. Red and blue indicate a high and low probability of occurrence, respectively.

By overlaying the distribution results from MaxEnt with those from Dark Diversity, we could find out that most of these dark plant potential regions were also their climatically suitable areas from current to 2070 in MaxEnt modeling. However, Jiangsu province was estimated having dark species *F. longipetiolata* while MaxEnt models predicted it not climatically suitable for *F. longipetiolata* either under current or future climatic scenarios (Fig. 4A).

In addition, for these six species, the projected climatic suitability in their potential regions would change under future climatic scenarios. Our MaxEnt modeling results showed that most species’ suitable areas would firstly increase by 2050, and then contract by 2070 (Fig. 4). However, *E. ulmoides* suitable area in Jiangsu province would shrink by 2050 and then expand a little by 2070 (Fig. 4A). We believed these shifts probably related to BIO14 decline from current to 2050, and BIO19 increase from current to 2070, and similar associations have also been found in other species (Bueno et al., 2017; Deb et al., 2017; Noulekoun et al., 2017; Wu et al., 2017).

## Discussion

Unlike most other studies to date, we estimated dark diversity using data at the provincial scale instead of at spatial grid (Lewis, Szava-Kovats & Pärtel, 2016; Ronk, Szava-Kovats & Pärtel, 2015). We do consider that a provincial level study is rather coarse in terms of its spatial scales, but still, the results of this study provide the first ever dark diversity patterns concentrated in China. A more robust study can be carried out when data at finer scales become available. Moreover, our result showed a similar distribution compared with the previous global scale study, where data at spatial grid were applied (Pärtel, Szava-Kovats & Zobel, 2011). Thirdly, gridded data at a finer scale may not always be able to bring more accuracy to dark diversity estimation. In the study by De Bello et al. (2016), they merged plot-level species lists into 18 habitats at a larger scale and produced habitat-level estimates of dark diversity estimates. They concluded that habitat-level analysis had a higher similarity with expert estimates than that in the grid (De Bello et al., 2016).

In this study, the results combination of dark diversity and Maxent helped six threatened species to find out their possibly suitable areas presently and in the future, and we believe it is valuable for ex situ conversation activities. Currently, the most common approach, that is, used in ex situ conservation is to collect rare species and grow them in plantations or resource nurseries and to maintain their seed banks (Hawkins, Sharrock & Havens, 2008). However, this requires the reestablishment of plants, which currently has a low success rate globally (Polak & Saltz, 2011; Sheean, Manning & Lindenmayer, 2012). MaxEnt modeling has already been used in species reintroduction and ecological restoration studies (Angelieri et al., 2016; Ardestani et al., 2015; Hiers et al., 2012; Remya, Ramachandran & Jayakumar, 2015; Yang et al., 2013), but its combination with dark diversity modeling will allow more suitable species and broader habitat to be found for species conservation. In spite of that, this combination can be applied to research on species reintroduction, ecological restoration and preventing exotic invasion. For example, possibly suitable areas predication can reduce labor and financial costs and increase the success rate of reintroductions.

At the same time, we have noticed that sometimes the results from two models may have a little contradiction, and we think the reason could be different types of data subject they were using or the different theories they were based on. For example, in our study, *F. longipetiolata* is a dark species in Jiangsu province while MaxEnt showed low climatic suitability for it there (Fig. 4). In addition, MaxEnt was only trained with climatic variables, but some other variables not covered in this study, such as anthropogenic activities, invasive species, atmospheric carbon dioxide, downward radiation, and evolutionary history may also affect the species distribution (Cardinale et al., 2012; Doblas-Miranda et al., 2017; Woodward & Kelly, 2008). Moreover, as a present-background (present-only) based model, MaxEnt can only estimate the relative likelihood and probability, not probability of occurrence, which can be estimated with presence-absence and occupancy-detection models (Gomes et al., 2018; Guillera-Arroita et al., 2015), and this may explain the differences from the result of dark diversity. Moreover, the use of one climate model scenario maybe also a potential limitation.

## Conclusions

Dark diversity is a new and useful concept to indicate how many species hidden behind its observed species in a community (Pärtel, Szava-Kovats & Zobel, 2011, 2013). Like other studies, we mapped the results of dark diversity in China, which shows that more and more plants could be potentially distributed and restored in the east of China. In spite of that, we combined the results of dark diversity and MaxEnt, and predicted the climatically suitable areas presently and in the future for six threatened species, which can be valuable for their conversation. This combination can also make a contribution to restoration efforts and invasion risk assessment of alien species.

## Supplemental Information

10.7717/peerj.6731/supp-1 Supplemental Information 1 Bioclimatic variables description. These variable data were downloaded from WorldClim with 2.5-min. (of a longitude/latitude degree) spatial resolution (this is about 4.5 km at the equator). We obtained this dataset for current period from WorldClim 2.0, covering the period from 1960 to 1990. Those for 2050 were downloaded from WorldClim 1.4, referring the time span of 2041–2060. Data for 2070 were extracted from WorldClim 1.4, over the period of 2061–2080.

10.7717/peerj.6731/supp-2 Supplemental Information 2 Different dark diversities and their respective quantile probabilities. According to this table, we defined our quantile probability as 1% in order to explore a larger species pool.

10.7717/peerj.6731/supp-3 Supplemental Information 3 The occurrence distribution of six threatened dark species.

10.7717/peerj.6731/supp-4 Supplemental Information 4 Bioclimatic variables correlation analysis used for species distribution projection. (A) under current scenario. (B) in 2050s. (C) in 2070s.

10.7717/peerj.6731/supp-5 Supplemental Information 5 Data source for six threatened dark species.

10.7717/peerj.6731/supp-6 Supplemental Information 6 R code and data used in this study.

## Additional Information and Declarations

### Data Availability

The following information was supplied regarding data availability: The raw measurements are available in the Supplemental Files.
