# Supplementary material for: Throwing light on dark diversity of vascular plants in China: predicting the distribution of dark and threatened species under global climate change
Source: PeerJ. 2019 Apr 9;7:e6731. doi: 10.7717/peerj.6731 (PMC6461033; doi:10.7717/peerj.6731)
Supplement: Supplemental Information 1 — These variable data were downloaded from WorldClim with 2.5-min. (of a longitude/latitude degree) spatial resolution (this is about 4.5 km at the equator). We obtained this dataset for current period from WorldClim 2.0, covering the period from 1960 to 1990. Those for 2050 were downloaded from WorldClim 1.4, referring the time span of 2041–2060. Data for 2070 were extracted from WorldClim 1.4, over the period of 2061–2080. [file peerj-07-6731-s001.docx]

S1. Supplemental table

Table S1 Bioclimatic variables description

| Variables | Descriptions |
| --- | --- |
| BIO1 | Annual Mean Temperature |
| BIO2 | Mean Diurnal Range |
| BIO3 | Isothermality |
| BIO4 | Temperature Seasonality |
| BIO5 | Max Temperature of Warmest Month |
| BIO6 | Min Temperature of Coldest Month |
| BIO7 | Temperature Annual Range |
| BIO8 | Mean Temperature of Wettest Quarter |
| BIO9 | Mean Temperature of Driest Quarter |
| BIO10 | Mean Temperature of Warmest Quarter |
| BIO11 | Mean Temperature of Coldest Quarter |
| BIO12 | Annual Precipitation |
| BIO13 | Precipitation of Wettest Month |
| BIO14 | Precipitation of Driest Month |
| BIO15 | Precipitation Seasonality |
| BIO16 | Precipitation of Wettest Quarter |
| BIO17 | Precipitation of Driest Quarter |
| BIO18 | Precipitation of Warmest Quarter |
| BIO19 | Precipitation of Coldest Quarter |
