# Supplementary material for: Throwing light on dark diversity of vascular plants in China: predicting the distribution of dark and threatened species under global climate change
Source: PeerJ. 2019 Apr 9;7:e6731. doi: 10.7717/peerj.6731 (PMC6461033; doi:10.7717/peerj.6731)
Supplement: Supplemental Information 2 — According to this table, we defined our quantile probability as 1% in order to explore a larger species pool. [file peerj-07-6731-s002.doc]

| Region | Different dark diversities and their respective quantile probabilities | | |
| --- | --- | --- | --- |
|  | 1% | 5% | 10% |
| Hebei(Tianjin, Beijing) | 54 | 40 | 25 |
| Neimenggu | 113 | 85 | 60 |
| Jilin | 107 | 89 | 67 |
| Heilongjiang | 142 | 122 | 100 |
| Liaoning | 88 | 75 | 48 |
| Gansu(Ningxia) | 28 | 13 | 10 |
| Shaanxi | 66 | 17 | 6 |
| Shanxi | 114 | 78 | 52 |
| Shandong | 159 | 89 | 48 |
| Henan | 155 | 80 | 35 |
| Shanghai(Jiangsu) | 143 | 68 | 25 |
| Anhui | 115 | 76 | 29 |
| Zhejiang | 55 | 29 | 11 |
| Hubei | 111 | 59 | 39 |
| Sichuan(Chongqing) | 16 | 1 | 0 |
| Xinjiang | 0 | 0 | 0 |
| Qinghai | 215 | 133 | 81 |
| Tibet | 21 | 11 | 3 |
| Yunnan | 0 | 0 | 0 |
| Guizhou | 114 | 53 | 23 |
| Hunan | 122 | 84 | 47 |
| Jiangxi | 119 | 76 | 44 |
| Fujian | 135 | 90 | 42 |
| Taiwan | 5 | 0 | 0 |
| Guangxi | 32 | 20 | 9 |
| Guangdong(Hongkong, Macau) | 41 | 17 | 6 |
| Hainan | 102 | 39 | 14 |
