# Supplementary figures and images for: Throwing light on dark diversity of vascular plants in China: predicting the distribution of dark and threatened species under global climate change
Source: PeerJ. 2019 Apr 9;7:e6731. doi: 10.7717/peerj.6731 (PMC6461033; doi:10.7717/peerj.6731)

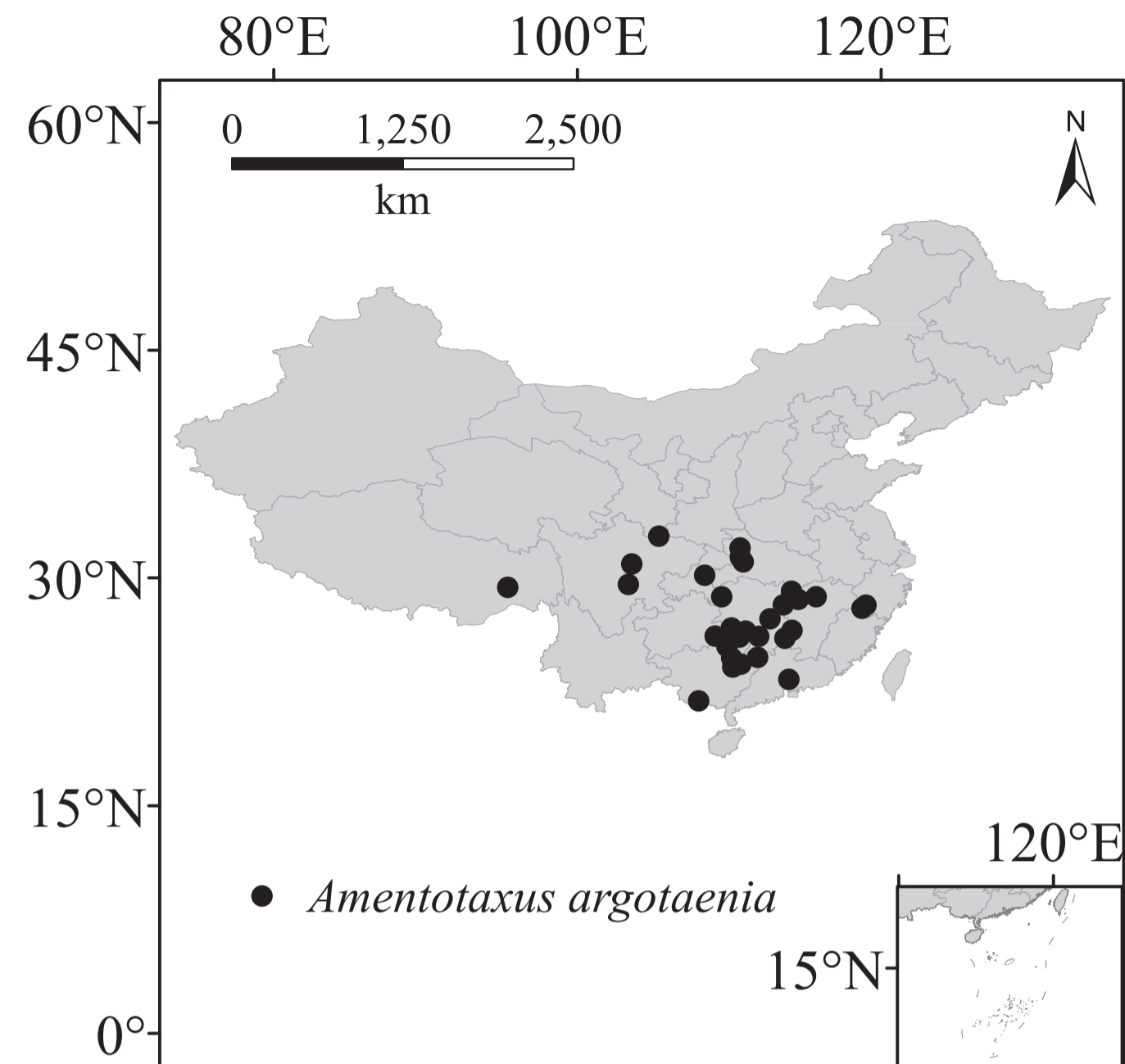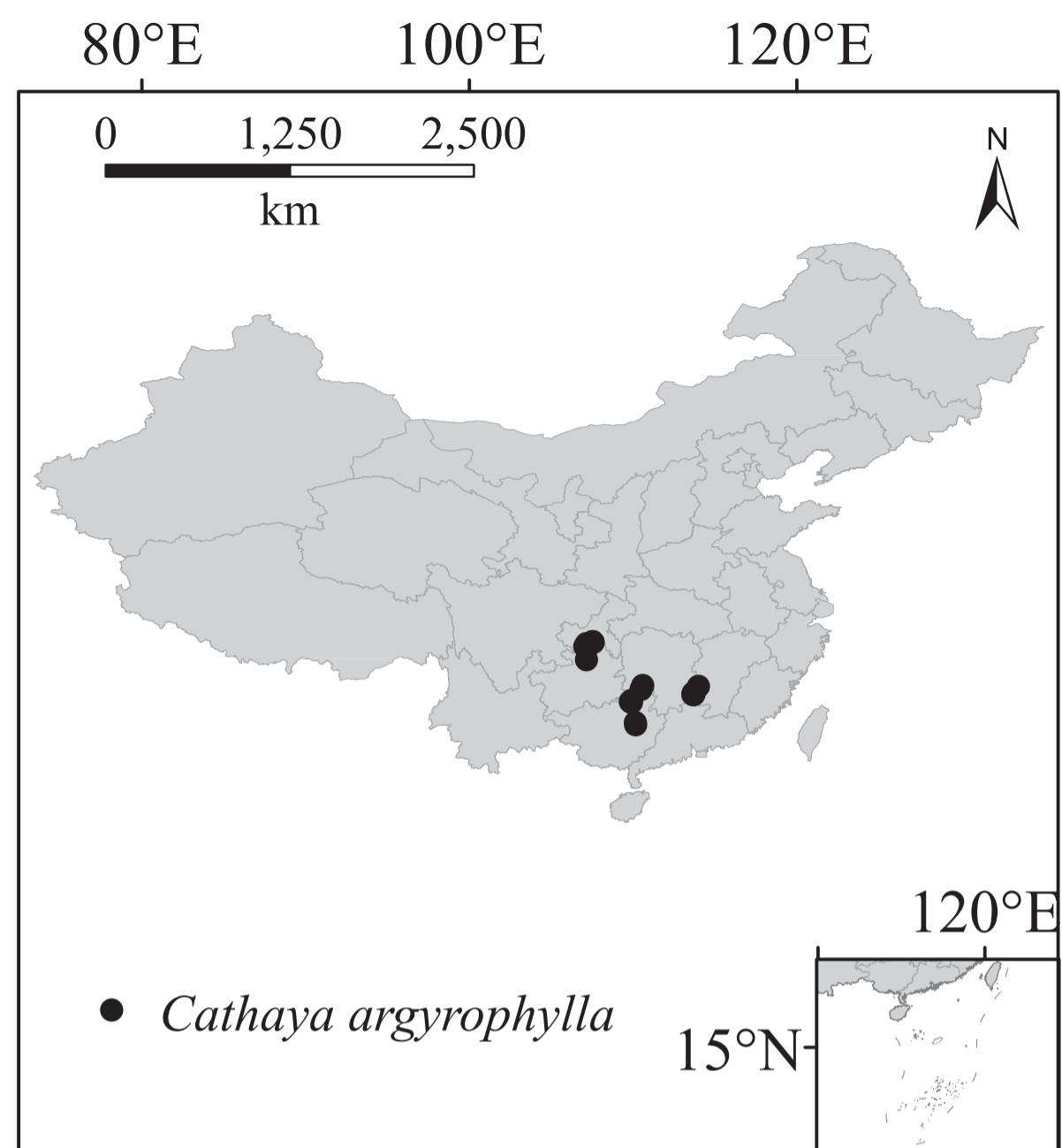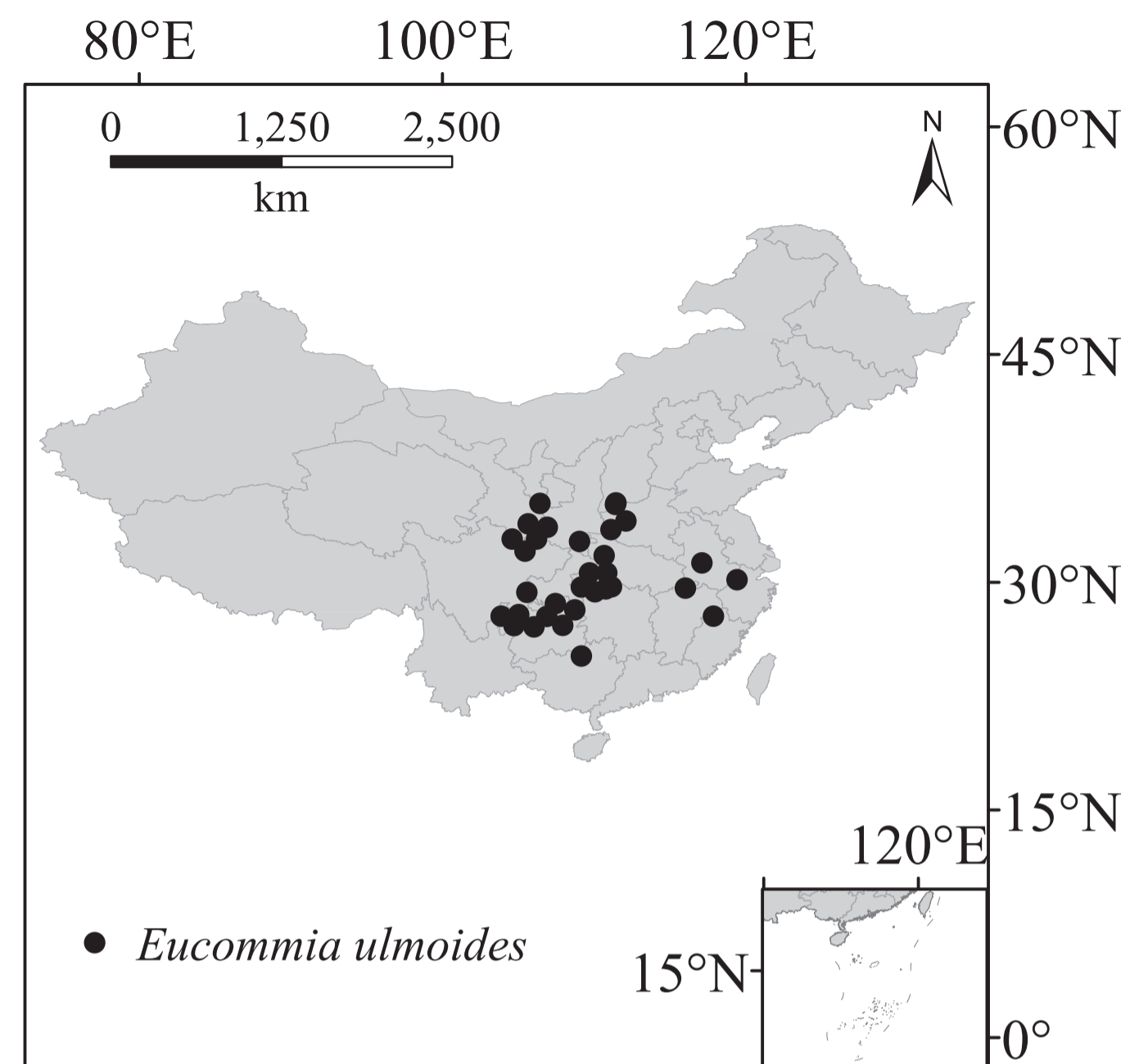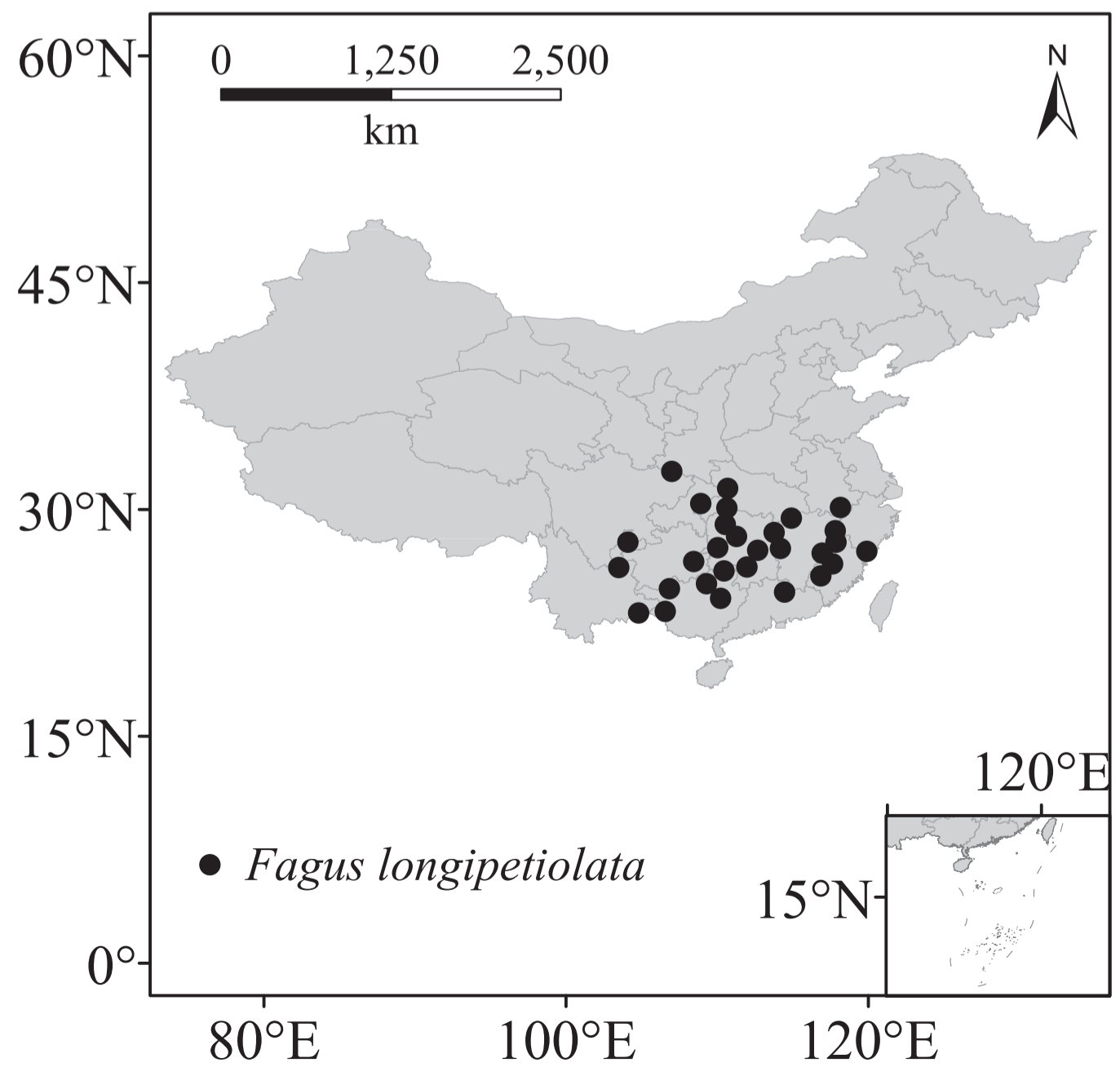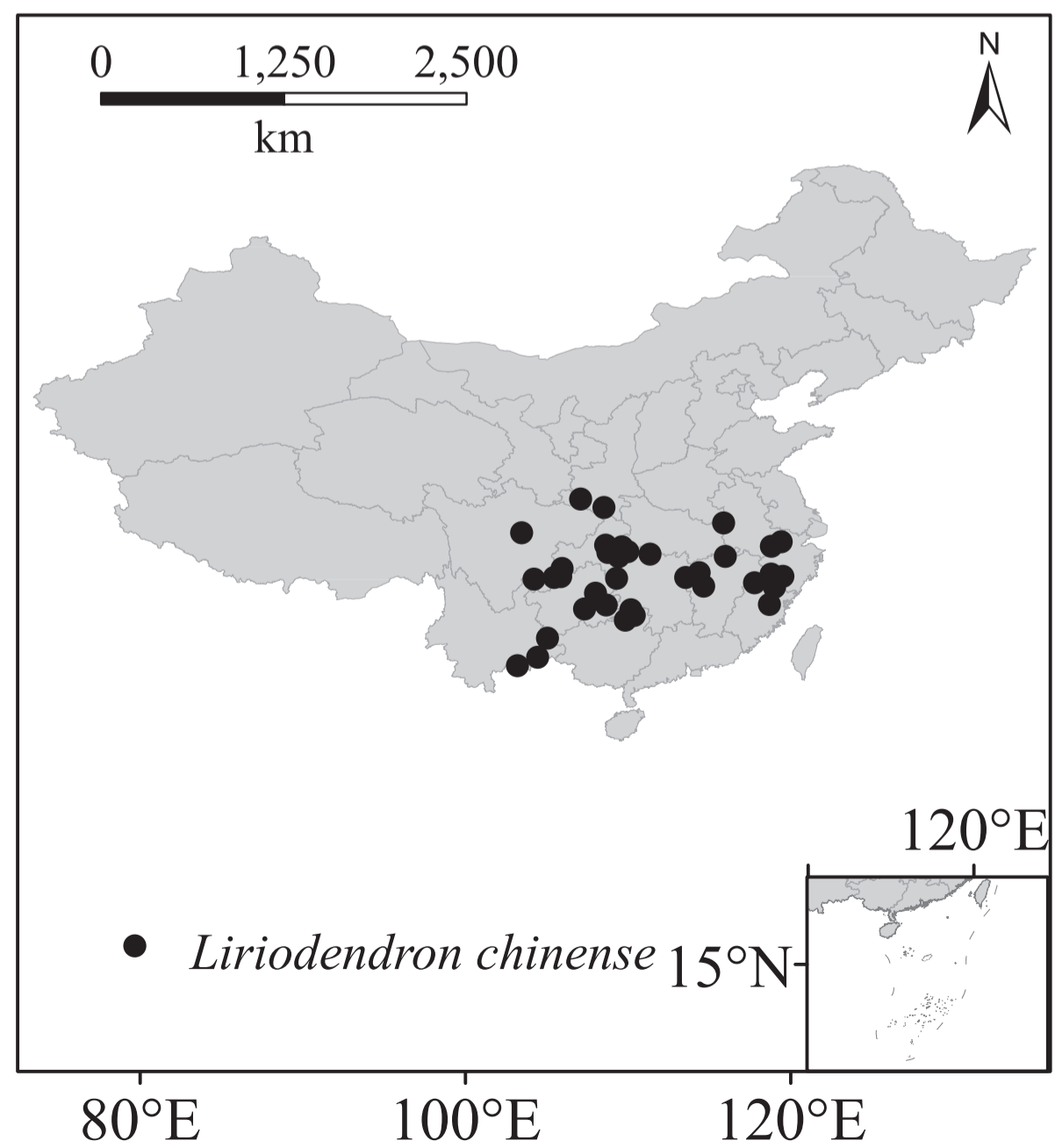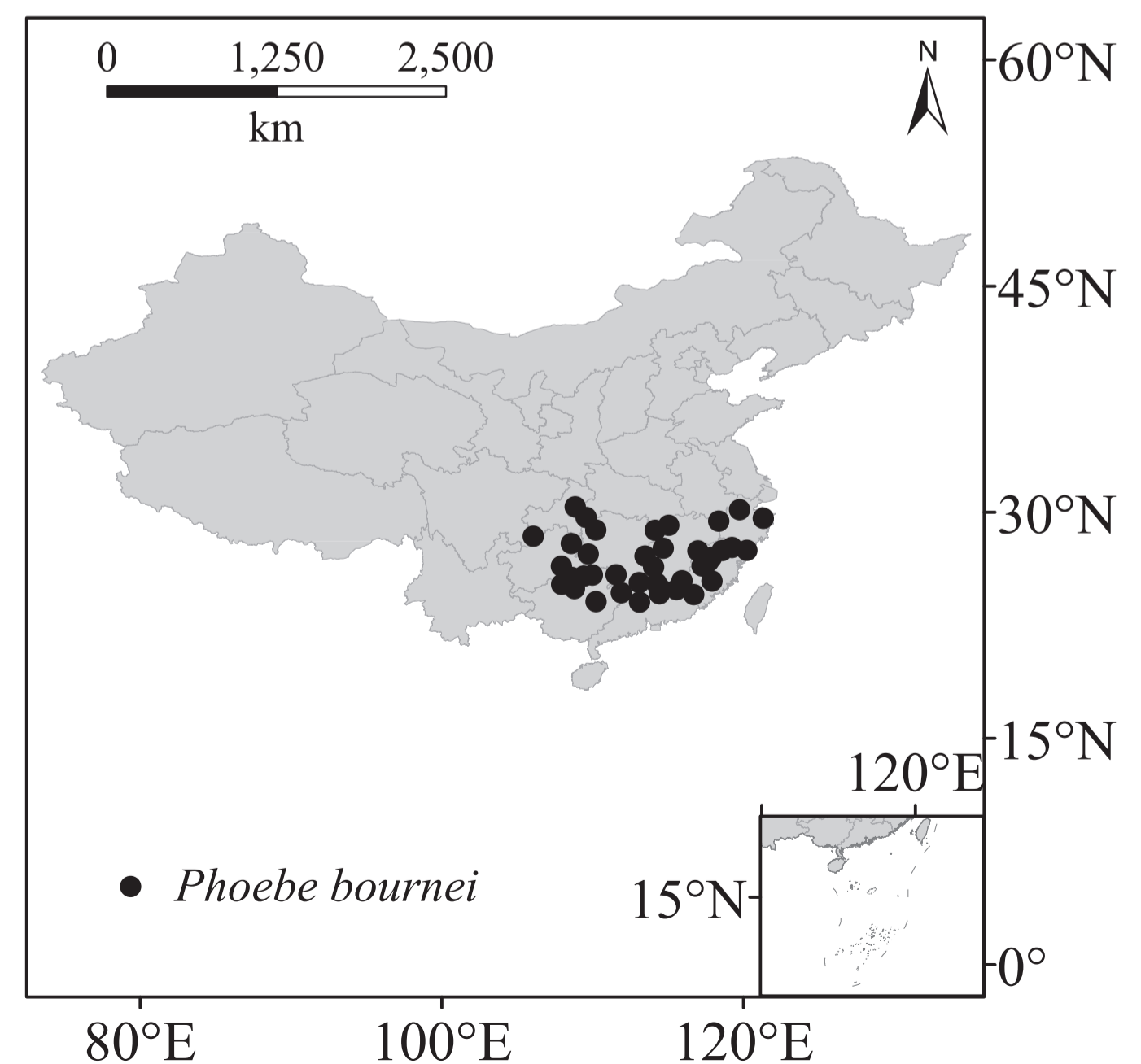

Supplement: Supplemental Information 3 [file peerj-07-6731-s003.pdf]

A

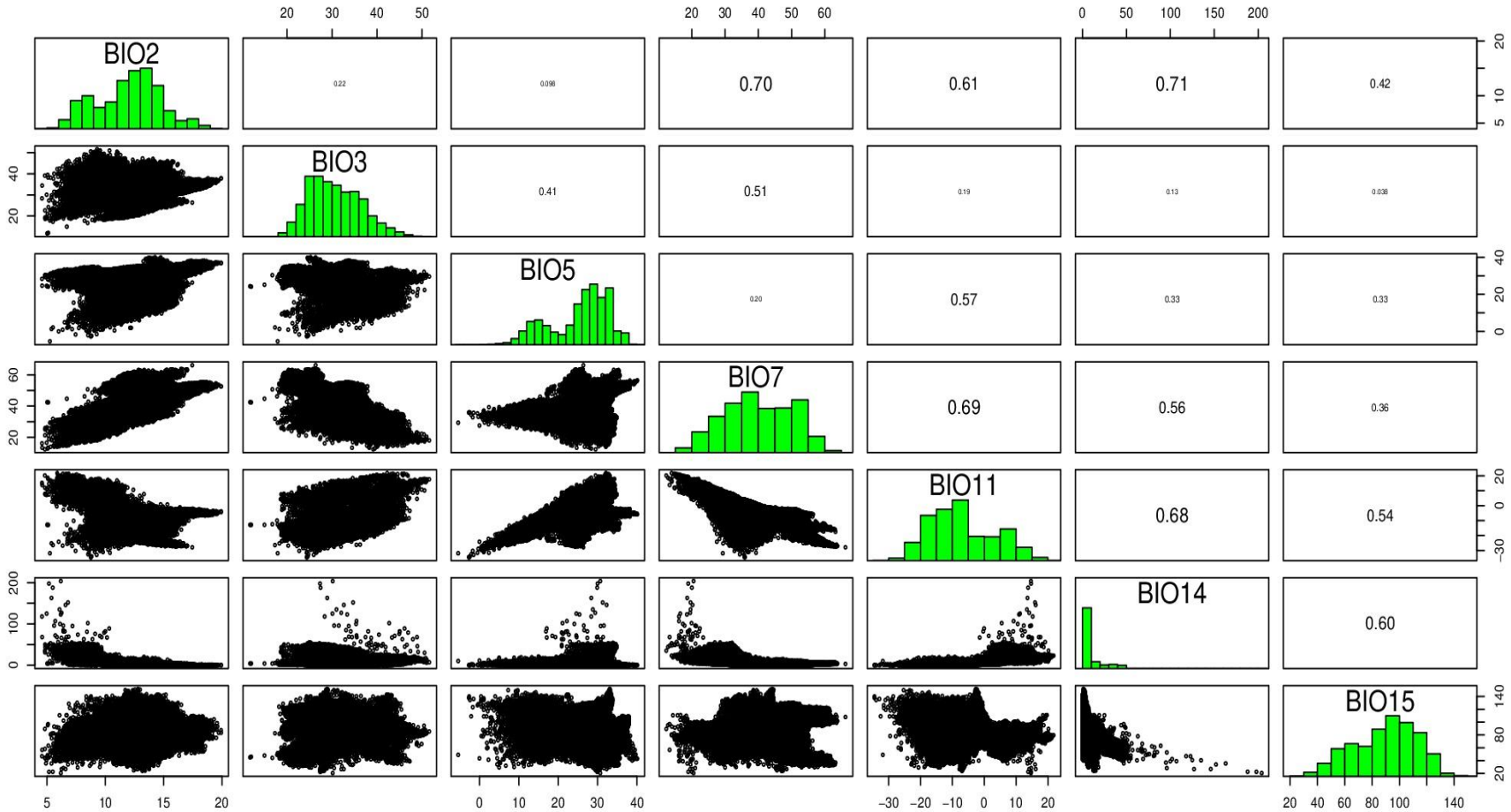

B

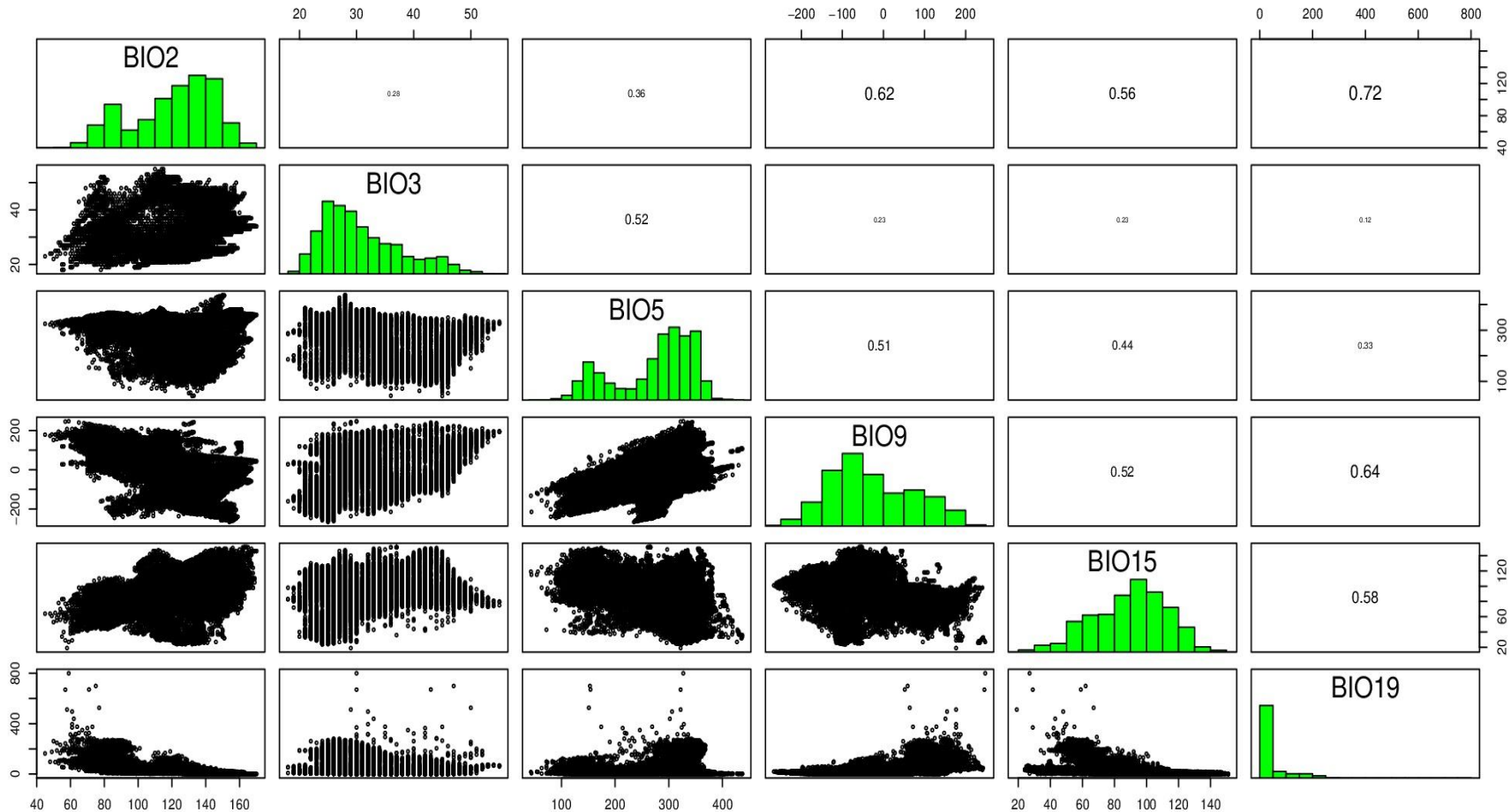

C

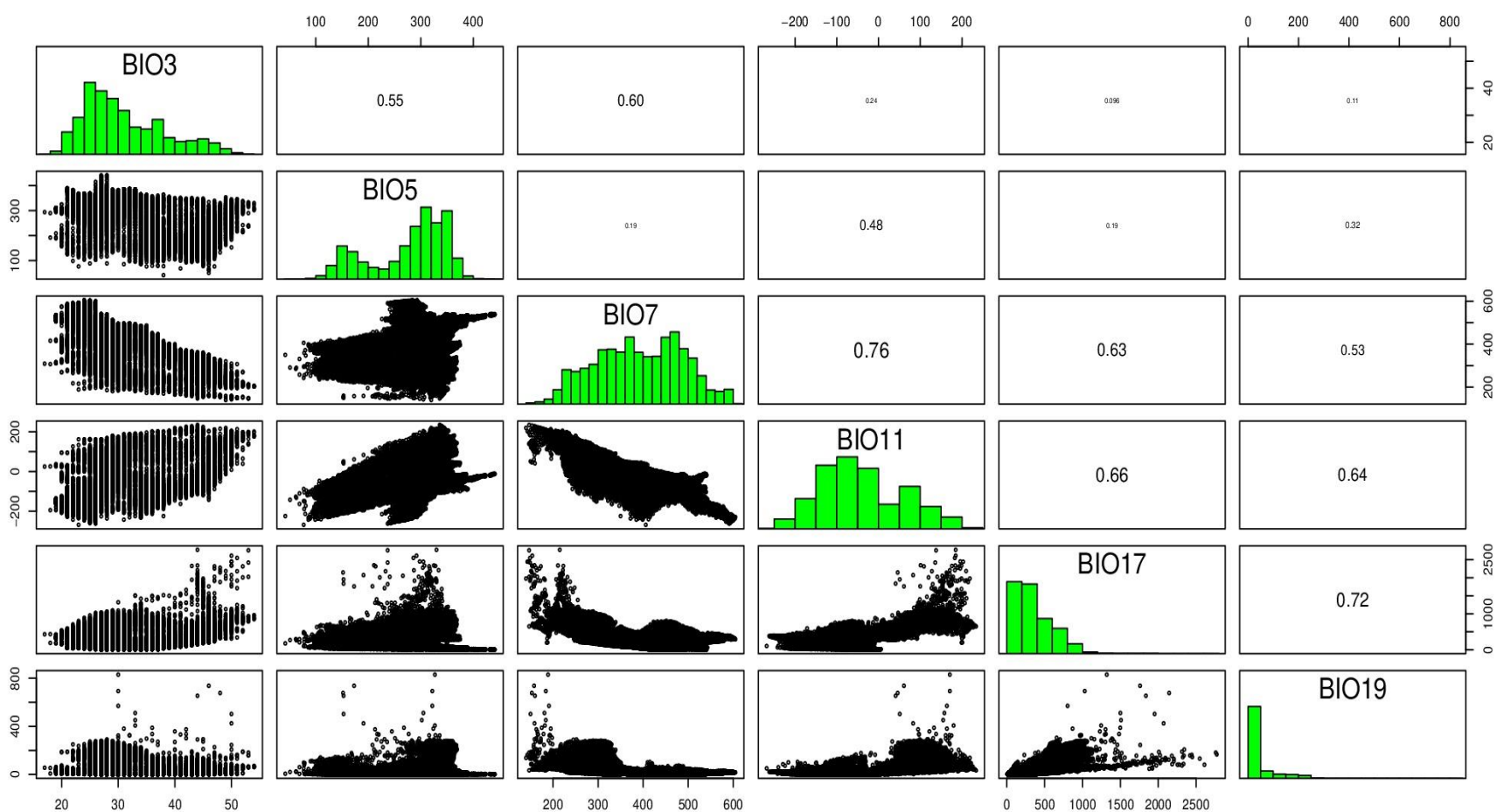

Supplement: Supplemental Information 4 — (A) under current scenario. (B) in 2050s. (C) in 2070s. [file peerj-07-6731-s004.pdf]
