# Supplementary material for: Throwing light on dark diversity of vascular plants in China: predicting the distribution of dark and threatened species under global climate change
Source: PeerJ. 2019 Apr 9;7:e6731. doi: 10.7717/peerj.6731 (PMC6461033; doi:10.7717/peerj.6731)
Supplement: Supplemental Information 5 [file peerj-07-6731-s005.doc]

[[1]](#endnote-2)Data source for MaxEnt

The presence records with geographic coordinates used in MaxEnt modelling

| Species | Longitude | Latitude |
| --- | --- | --- |
| *Amentotaxus argotaenia* | 110.226635 | 24.118302 |
|  | 110.143831 | 26.685143 |
|  | 111.87782 | 24.760847 |
|  | 113.666124 | 26.017481 |
|  | 109.076212 | 26.147546 |
|  | 114.105166 | 29.102876 |
|  | 114.554122 | 28.566211 |
|  | 103.574157 | 30.903249 |
|  | 118.999048 | 28.198925 |
|  | 118.750862 | 27.990568 |
|  | 105.355848 | 32.73756 |
|  | 110.734434 | 31.36327 |
|  | 113.926144 | 23.308511 |
|  | 109.852767 | 25.467659 |
|  | 110.722816 | 31.952092 |
|  | 114.142515 | 26.528603 |
|  | 115.719011 | 28.753391 |
|  | 110.930833 | 31.045612 |
|  | 108.377598 | 30.167185 |
|  | 103.355296 | 29.55816 |
|  | 109.504489 | 28.741671 |
|  | 111.074387 | 26.484524 |
|  | 112.687492 | 27.290972 |
|  | 111.940911 | 26.140285 |
|  | 113.562798 | 28.203972 |
|  | 95.415692 | 29.370283 |
|  | 110.661252 | 26.081036 |
|  | 110.156258 | 24.707495 |
|  | 110.762102 | 24.298548 |
|  | 107.981964 | 21.887605 |
|  | 110.226635 | 24.118302 |
|  | 110.143831 | 26.685143 |
|  | 111.87782 | 24.760847 |
|  | 113.666124 | 26.017481 |
|  | 109.076212 | 26.147546 |
|  | 114.105166 | 29.102876 |
|  | 114.554122 | 28.566211 |
|  | 103.574157 | 30.903249 |
|  | 118.999048 | 28.198925 |
|  | 118.750862 | 27.990568 |
|  | 105.355848 | 32.73756 |
|  | 110.734434 | 31.36327 |
|  | 113.926144 | 23.308511 |
|  | 109.852767 | 25.467659 |
|  | 110.722816 | 31.952092 |
|  | 114.142515 | 26.528603 |
|  | 115.719011 | 28.753391 |
|  | 110.930833 | 31.045612 |
|  | 108.377598 | 30.167185 |
|  | 103.355296 | 29.55816 |
|  | 109.504489 | 28.741671 |
|  | 111.074387 | 26.484524 |
|  | 112.687492 | 27.290972 |
|  | 111.940911 | 26.140285 |
|  | 113.562798 | 28.203972 |
|  | 95.415692 | 29.370283 |
|  | 110.661252 | 26.081036 |
|  | 110.156258 | 24.707495 |
|  | 110.762102 | 24.298548 |
|  | 107.981964 | 21.887605 |
| *Cathaya argyrophylla* | 113.708858 | 26.066667 |
|  | 107.561436 | 29.192535 |
|  | 107.592712 | 29.135619 |
|  | 107.550227 | 29.183933 |
|  | 109.849646 | 25.536005 |
|  | 109.91857 | 25.48845 |
|  | 107.195298 | 28.797504 |
|  | 110.182931 | 24.108739 |
|  | 114.022702 | 26.46202 |
|  | 110.608889 | 26.519444 |
|  | 107.170893 | 28.092315 |
|  | 113.669 | 25.961 |
|  | 113.729 | 25.951 |
|  | 113.76 | 26.033 |
|  | 107.067 | 28.9 |
|  | 107.167 | 29.067 |
|  | 107.2 | 28.883 |
|  | 107.551 | 29.171 |
|  | 110.169 | 24.253 |
|  | 109.911 | 25.581 |
|  | 109.915 | 25.604 |
|  | 110.506517 | 26.271963 |
| *Eucommia ulmoides* | 110.770996 | 29.539252 |
|  | 106.438789 | 35.178332 |
|  | 104.600017 | 32.823008 |
|  | 105.651544 | 33.817658 |
|  | 106.207568 | 32.821843 |
|  | 109.039021 | 32.66186 |
|  | 106.91284 | 33.600938 |
|  | 111.43986 | 35.209046 |
|  | 111.413767 | 35.096375 |
|  | 112.089943 | 34.009572 |
|  | 111.119525 | 33.443796 |
|  | 109.158986 | 29.65922 |
|  | 109.692477 | 30.595326 |
|  | 110.834959 | 30.602892 |
|  | 110.661458 | 31.736289 |
|  | 105.44478 | 32.028596 |
|  | 103.878482 | 27.746699 |
|  | 105.02996 | 27.85456 |
|  | 109.155244 | 25.11529 |
|  | 117.110377 | 31.251809 |
|  | 117.874138 | 27.737671 |
|  | 105.569091 | 29.308775 |
|  | 111.142025 | 29.673174 |
|  | 110.055361 | 29.344778 |
|  | 108.726294 | 28.1549 |
|  | 116.013107 | 29.59299 |
|  | 107.4467 | 28.5745 |
|  | 106.8793 | 27.7262 |
|  | 106.035 | 27.0291 |
|  | 107.9336 | 27.1377 |
|  | 104.7281 | 27.124 |
|  | 119.430107 | 30.140757 |
| *Fagus longipetiolata* | 110.643485 | 30.081465 |
|  | 110.705629 | 31.380335 |
|  | 108.919694 | 30.382959 |
|  | 108.449099 | 26.550442 |
|  | 110.457114 | 25.916657 |
|  | 110.228451 | 24.109985 |
|  | 106.851691 | 24.750244 |
|  | 119.911246 | 27.218481 |
|  | 117.640205 | 26.384238 |
|  | 116.864731 | 25.595112 |
|  | 114.465305 | 24.520373 |
|  | 117.821859 | 28.579816 |
|  | 116.946669 | 27.098589 |
|  | 114.912477 | 29.408951 |
|  | 114.18557 | 27.409536 |
|  | 118.170213 | 30.113491 |
|  | 104.094636 | 27.815928 |
|  | 103.502743 | 26.138883 |
|  | 104.811776 | 23.131912 |
|  | 112.687255 | 27.286176 |
|  | 111.282987 | 28.199712 |
|  | 111.982971 | 26.175209 |
|  | 110.050139 | 27.454509 |
|  | 107.000733 | 32.500406 |
|  | 110.535604 | 29.003661 |
|  | 109.294916 | 25.083309 |
|  | 106.574392 | 23.250028 |
|  | 117.868638 | 27.819519 |
|  | 113.771117 | 28.463557 |
| *Liriodendron chinense* | 118.832333 | 30.123167 |
|  | 115.905666 | 31.559495 |
|  | 118.719349 | 26.547022 |
|  | 117.804167 | 27.881167 |
|  | 109.85 | 25.583333 |
|  | 105.067544 | 24.462907 |
|  | 110.397233 | 25.873683 |
|  | 107.35 | 26.266667 |
|  | 108.672 | 26.501833 |
|  | 109.319667 | 28.154667 |
|  | 108.026889 | 27.232228 |
|  | 105.887544 | 28.253477 |
|  | 109.641667 | 30.0875 |
|  | 108.7815 | 29.744361 |
|  | 110.014983 | 29.788753 |
|  | 108.649356 | 30.184571 |
|  | 110.19 | 26.22 |
|  | 113.560605 | 28.205842 |
|  | 111.379486 | 29.631887 |
|  | 109.439199 | 29.479813 |
|  | 115.997771 | 29.500279 |
|  | 114.67405 | 27.666101 |
|  | 114.4 | 28.5 |
|  | 108.531648 | 32.497656 |
|  | 107.098636 | 33.016751 |
|  | 105.486833 | 28.196667 |
|  | 105.973916 | 28.761797 |
|  | 103.479942 | 30.943115 |
|  | 103.2255 | 22.777 |
|  | 104.241937 | 28.106093 |
|  | 104.465 | 23.295333 |
|  | 119.432667 | 30.404167 |
|  | 119.55 | 28.27 |
|  | 119.019716 | 27.597536 |
|  | 118.827 | 28.411 |
| *Phoebe bournei* | 119.777276 | 30.157973 |
|  | 121.344957 | 29.583277 |
|  | 115.073553 | 29.111263 |
|  | 114.174959 | 28.782343 |
|  | 114.712638 | 27.596746 |
|  | 116.998702 | 27.422572 |
|  | 117.903581 | 26.975254 |
|  | 120.250473 | 27.464378 |
|  | 116.721893 | 24.52205 |
|  | 115.580894 | 24.852774 |
|  | 115.947362 | 25.445721 |
|  | 114.572756 | 24.867554 |
|  | 113.130779 | 24.017311 |
|  | 114.263655 | 25.285046 |
|  | 113.117956 | 25.308947 |
|  | 114.062198 | 26.358501 |
|  | 113.495052 | 27.073037 |
|  | 111.586685 | 25.83066 |
|  | 109.731931 | 27.216982 |
|  | 110.224599 | 28.800908 |
|  | 109.58593 | 29.635184 |
|  | 108.871931 | 30.338194 |
|  | 108.603003 | 27.906444 |
|  | 107.956899 | 26.407087 |
|  | 108.621607 | 25.669204 |
|  | 107.969261 | 25.202925 |
|  | 110.008815 | 25.82615 |
|  | 108.815418 | 24.92329 |
|  | 110.256611 | 24.063049 |
|  | 106.092666 | 28.395803 |
|  | 111.926553 | 24.640859 |
|  | 117.718391 | 26.709456 |
|  | 118.379 | 29.396 |
|  | 114.440415 | 24.580758 |
|  | 117.278 | 26.418 |
|  | 109.44 | 25.753 |
|  | 117.933 | 25.426 |
|  | 118.616711 | 27.442677 |
|  | 119.265 | 27.655 |

Reference:

Amentotaxus argotaenia:

1 ZHOU M, LI BQ, LIU KW. (2007), Study on Biological and Ecological Characteristics of Endangered Plants Amentotaxus angotaenia. *Forest inventory and planning*, 32: 136-139.

2 ZHONG MJ, YE XB, LIAO WB. (2004), Study on vascular plants flora from Xinhui regions in Guangdong province,China. *Acta botanica boreali-occidentalia sinica*, 23: 1246-1257.

3 PENG QQ, WANG ZX, TANG Y, CHEN Y, HAN X. (2017), Quantitative Assessment of Priority for Rare andEndangered Plants in Yerengu Nature Reserve of Hubei. *Forest resources management*, 2: 143-150.

4 WANG L, SHI S, LIAO WB, CHEN CQ, LI Z. (2013), Rare and endangered plants in Mount Jinggangshan region. *Biodiversity science*, 21: 163-169.

5 LIU KW, XUE SG, XIAO QD. (2001), The Succession of Amentotaxus argotaenia Community. *Journal of central south forestry university*, 21: 23-27.

6 SHEN ZH, JIN YX, ZHAO ZE, WU JQ, HUANG HD. (2000), The structure and dynamics of the rareplant communities in subtropical mountian of China. *Acta ecological sinica*, 20: 800-807.

Eucommia ulmoides：

7 KANG CZ, WANG QQ, ZHOU T, JIANG WK, XIAO CH, XIE Y. (2014), Study on Ecological Suitability Regionalization of Eucommia ulmoides in Guizhou. Journal of Chinese medicinal materials, 37: 760-766.

8 ZHOU Q, CHEN GX, XIONG LZ, HUANG M, LIU C, CAI ZX. (2014), Diversity of samara characters of Euocmmia ulmoides in western of Hunan. Journal of central south University of forestry and technology, 4: 14-19.

9 HU AH. (2016), Study on distribution and change of Magnolia officinalis and Eucommia Ulmoides original in Yu-E-Xiang-Qian bounded neighbour region from Tang to Qing Dynasties. Chinese wild plant resources, 35: 46-48,52.

10 WANG XY, ZHU XM, WANG Q, SU QL, KANG XY, WU SZ, ZHOU MS, ZHANG LF, CAO CP. (2013), Establishment of ISSR-PCR reaction system, primers screening and their applications to genetic diversity analysis in Eucommia ulmoides. Nonwood forest research, 31: 30-34.

Liriodendron chinense:

11 YANG AH, ZHANG JJ, TIAN H, YAO XH, HUANG HW. (2014), Microsatellite genetic diversity and fine-scale spatial genetic structure within a natural stand of Liriodendron chinense (Magnoliaceae) in Lanmushan, Duyun City, Guizhou Province. Biodiversity science, 22: 375-384.

12 Li, K., Chen, L., Feng, Y., Yao, J., Li, B., Xu, M., & Li, H. (2014). High genetic diversity but limited gene flow among remnant and fragmented natural populations of Liriodendron chinense Sarg. Biochemical systematics and ecology, 54, 230-236.

13 TU CY, FU ZL, ZHAO D, DING QD, QI H, LIAN E. (2016). The Flora of Woody Plants in Bailongjiang and Taohe Forest Regions, Gansu province. Practical Forestry Technology, 10: 8-13.

14 CHAI FX, ZOU TF, CHEN XN. (2003). Protection and utilization of nationally preserved emphasis wild plants in Gansu Province. Chinese Journal of Ecology, 22:94-97.

15 ZHANG L, XIE SX, WU ZW, WANG YF. (2011). The Similarity and Cluster Analysis of Forest Communities in Xishui National Nature Reserve of Guizhou Province. Guizhou Agricultural Sciences, 39: 170-172,177.

16 LI B, LIU HX. (2013). Spatial Pattern Analysis of Individuals in Different Age Classes of Liriodendron chinensis Sarg in Maoer Mountain Reserve. Northern Horticulture, 21: 81-85.

17 FANG YP, LIU SX, WANG ZX, LEI Y, MAN JS. (2007). Quantitative Assessment of Priority for Conservation of the National Protected Plants in Qizimeishan Mountain Nature Preserve. Acta Botanica Boreali-occidentalia Sinica, 27: 348-355.

18 Yang, A., Dick, C. W., Yao, X., & Huang, H. (2016). Impacts of biogeographic history and marginal population genetics on species range limits: a case study of Liriodendron chinense. Scientific reports, 6, 25632.

Phoebe bournei :

19 GE YJ, WANG JF, FANG W, YE RH. (2012), Distribution Pattern of Phoebe bournei (Hemsl.) and the Characteristics of Climate. Acta Agriculturae Universitatis Jiangxiensis (Natural Sciences Edition), 34: 749-753,761.

Cathaya argyrophylla :

20 SU LY, ZHAO WY, ZHANG JJ, YANG YP, GUO YF, FAN Q, LIAO WB. (2016), Analyses on community characteristics and its relict and conservation of Cathaya argyrophylla at Bamianshan in Hu’ nan Province. Journal of plant resources and environment, 25: 76-86.

21 WANG, H. and GE, S. (2006), Phylogeography of the endangered Cathaya argyrophylla (Pinaceae) inferred from sequence variation of mitochondrial and nuclear DNA. Molecular Ecology, 15: 4109-4122.

22 XIE ZQ, CHEN WL. (1994), The Present status and the future of Cathaya Argyrophylla forest. Chinese Biodiversity, 1: 11-15.

Fagus longipetiolata:

23 ZHANG CG, XU HX, JIANG QN. (1997), Distribution, growth and revegetational characteristics of Fagus in China. Chinese Journal of Ecology, 4: 48-52.

24 LI TF, LI JQ. (2008), The Origination, distribution, regeneration and genetic diversity of Chinese beech. Chinese Agricultural science bulletin, 24: 185-191.

1. [↑](#endnote-ref-2)
